# Cohort-specific boolean models highlight different regulatory modules during Parkinson’s disease progression

**DOI:** 10.1016/j.isci.2024.110956

**Published:** 2024-09-14

**Authors:** Ahmed Abdelmonem Hemedan, Venkata Satagopam, Reinhard Schneider, Marek Ostaszewski

**Affiliations:** 1 Bioinformatics Core Unit, Luxembourg Centre for Systems Biomedicine, University of Luxembourg, Esch-sur-Alzette, Luxembourg

**Keywords:** Biological sciences, Bioinformatics, Computational bioinformatics

## Abstract

Parkinson’s disease (PD) involves complex molecular interactions and diverse comorbidities. To better understand its molecular mechanisms, we employed systems medicine approaches using the PD map, a detailed repository of PD-related interactions and applied Probabilistic Boolean Networks (PBNs) to capture the stochastic nature of molecular dynamics. By integrating cohort-level and real-world patient data, we modeled PD’s subtype-specific pathway deregulations, providing a refined representation of its molecular landscape. Our study identifies key regulatory biomolecules and pathways that vary across PD subtypes, offering insights into the disease’s progression and patient stratification. These findings have significant implications for the development of targeted therapeutic interventions.

## Highlights

• Boolean models identify distinct pathways in Parkinson’s disease subtypes
• Dysregulated pathways include dopamine signaling, mitochondrial function, and autophagy
• Type 2 diabetes worsens mitochondrial dysfunction and neuron survival in Parkinsonism
• Subtype-specific models suggest targets for personalized Parkinson’s disease treatments

## Graphical abstract

**Figure:**
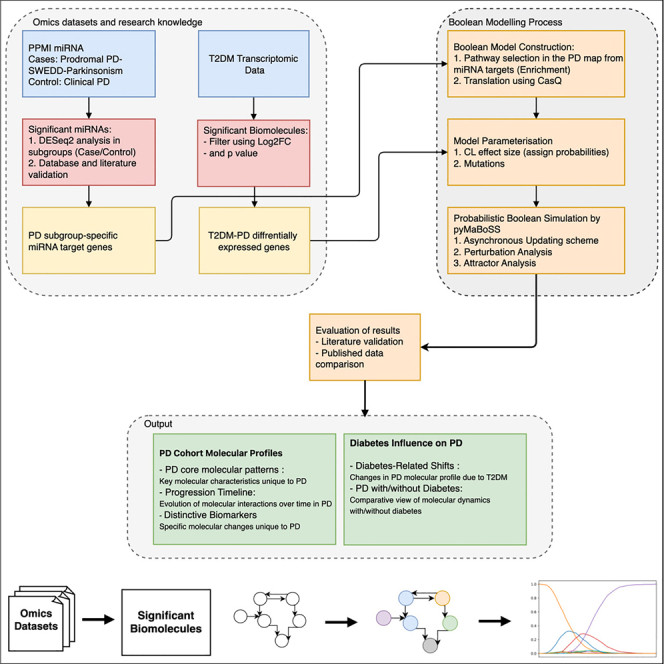

## Introduction

Parkinson’s disease (PD) is a complex disorder characterized by the progressive degeneration of dopaminergic neurons. This degeneration leads to a variety of motor and cognitive impairments.^1^ PD is often accompanied by various comorbidities, such as dementia and diabetes,^2^ Which further complicates its clinical symptoms and management. An in-depth understanding of PD complexity necessitates a systematic analysis and interpretation of its various subtypes and associated conditions.^3^

An important aspect of PD is its molecular pathophysiology. Neuronal degeneration in PD, particularly in the substantia nigra, is closely linked to disruptions in dopamine release, affecting motor cortex stimulation. This molecular perspective is crucial for understanding the disease’s progression and symptoms, such as muscular rigidity, tremors, and bradykinesia.^4^ Moreover, the role of molecular mechanisms in the pathogenesis and progression of PD, especially in the context of its comorbidities, remains an important area of exploration.

To investigate the complex interplay of molecular mechanisms in PD, systems biomedicine approaches can be employed.^5^^,^^6^^,^^7^ These approaches allow for a comprehensive analysis of the disease at a molecular level, integrating various biological data and computational methods. By examining the molecular pathways and their interactions, systems biomedicine provides a holistic view of the disease’s underlying mechanisms.^8^ These approaches enable dynamic analysis of complex molecular networks, crucial for understanding PD progression. This necessitates encoding detailed knowledge and relies on a robust simulation framework for accuracy.

In our approach, we utilize PD map^9^ as a primary knowledge source and logical modeling as a computational paradigm.^10^ The PD map encapsulates extensive knowledge about PD-related mechanisms, offering a crucial tool for visualizing and understanding molecular interactions implicated in the disease. Logical modeling complements this by providing both qualitative and quantitative analyses of disease mechanisms, enabling a deeper understanding of the complex biological systems involved in PD. In our approach, we use the PD map as a knowledge source of PD mechanisms.^9^ The PD map represents the largest repository of PD pathways available to date, including detailed knowledge about PD-related mechanisms. It serves as an essential tool for visualizing molecular interactions implicated in the disease. The pathway diagrams in the PD map are static but can be modeled and simulated to understand the dynamics of the represented mechanisms.^11^

To accurately model the subtype-specific pathway deregulation in PD, we incorporate cohort and real-world omics data. This integration allows us to examine the heterogeneity of the disease and the specific molecular responses to various perturbations. By integrating this data, our models become more representative of the disease mechanisms in different patient groups. To combine logical modeling with empirical data, we rely on Probabilistic Boolean Networks (PBNs).^10^ PBNs enable us to simulate the impact of molecular dysregulation, thus providing more understanding of the disease pathways. This approach is particularly effective in exploring the complexities of PD pathways and their variations across different disease subtypes.^12^^,^^13^

Our analysis identified differentially expressed miRNAs, ensuring they were validated and had established interactions in brain tissue. Significant miRNAs showed downregulation in PD and were involved in mitochondrial dysfunction. The enrichment analysis using the PD map highlighted key pathways potentially involved in disease progression. These pathways were converted into dynamic probabilistic Boolean models. Building upon the miRNA data, these models were parameterized to represent the disease cohorts. These models revealed distinct behaviors in key molecular pathways across PD subtypes, including dopamine transcription, PI3K/AKT signaling, FOXO3 activity, mTOR-MAPK signaling, and PRKN mitophagy. There was significant dysregulation in mitochondrial biogenesis and neuron survival in the Parkinsonism group, which was exacerbated by Type 2 diabetes mellitus (T2DM). Additionally, our study revealed notable variations in insulin resistance patterns, with the Prodromal group displaying distinctly different profiles compared to other PD subtypes. differences in autophagy and mitophagy activities were observed, suggesting unique disease mechanisms within each PD subtype. This understanding of PD subtypes and the impact of T2DM comorbidity can help to develop targeted and personalized treatment approaches.

This article is structured as follows: in the next Section, we provide an outline of the fundamental aspects of PD, its molecular mechanisms, and the methodologies employed in this study. The next Section presents methods and results of our study of cohort-specific dynamics in key PD pathways. Finally, we discuss our results and conclude with reflections on the potential impact of our research and directions for future studies in this field.

### Background

Parkinson’s disease (PD) is a complex, chronic, and age-related disorder. PD is characterized by the accumulation of alpha-synuclein proteins, leading to the formation of Lewy bodies, which are central to neuronal degeneration.^14^ This is complicated by mitochondrial dysfunctions, which disrupt cellular energy production, and oxidative stress that damages cellular structures.^15^ Further, neuroinflammation contributes to the progressive nature of disease by increasing neural loss.^16^ These mechanisms are interconnected and affect each other, which increases the disease complexity.^1^

The complexity of PD is not limited to its underlying mechanisms. The disease’s interaction with comorbid conditions, such as Type 2 diabetes mellitus, introduces an additional layer of complexity. Studies show that T2DM can exacerbate mitochondrial dysfunction in PD, leading to an accumulation of metabolic byproducts that lead to neuronal death.^17^ Further, the use of antidiabetic drugs has shown promise as neuroprotective agents in PD, suggesting common therapeutic pathways for these comorbid conditions.^18^ Moreover, studies highlight the interplay between α-synuclein pathology, a hallmark of PD, and metabolic dysfunctions characteristic of T2DM, linking these conditions at a molecular level.^19^ The complex relationship between PD and its comorbidity highlights the need for a comprehensive cohort data to investigate the diverse and intricate subtypes of this multifaceted disease.

**Figure 1:**
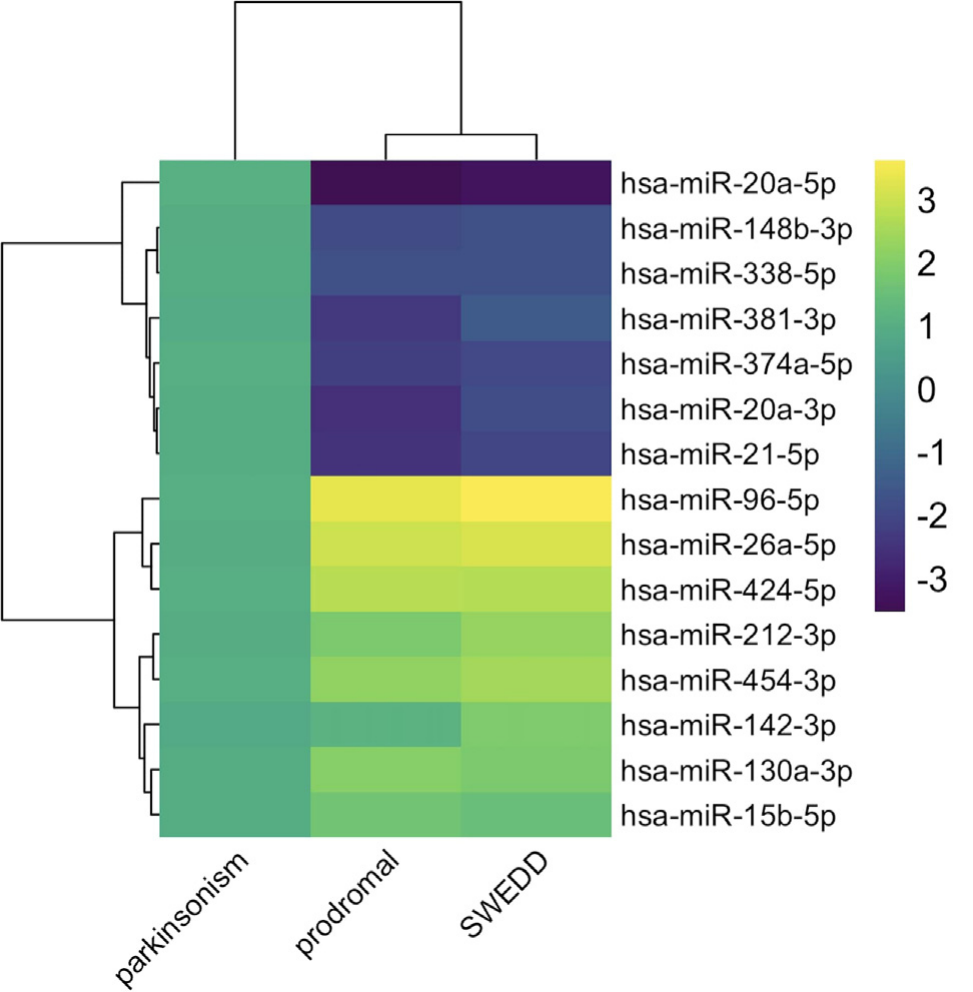
Significant miRNAs with Differential Expressions The figure displays significant miRNAs that are common across various conditions. his heatmap represents the differential expression of selected miRNAs across three groups: Parkinsonism, Prodromal, and SWEDD. The color gradient from purple to yellow indicates expression levels, with purple representing downregulation (−3) and yellow representing upregulation (+3). miRNAs such as hsa-miR-20a-5p and hsa-miR-148b-3p show significant differential expression, providing insights into miRNA involvement across various conditions.

The diversity of PD is reflected in its various subtypes, each with unique molecular signatures. Analyzing cohort-level data, such as from the Parkinson’s Progression Markers Initiative (PPMI) cohort study, can help to study the complexities of these subtypes.^20^ This approach enables the identification of specific biomarkers in each subtype, allowing for the developing of precise treatment in PD. The PPMI study provides a rich dataset for analyzing PD subtypes, focusing on prodromal, SWEDD (Scans Without Evidence of Dopaminergic Deficit), and parkinsonism cohorts. The prodromal stage represents early PD signs before clear motor symptoms appear. Patients with SWEDD exhibit PD-like symptoms but lack dopaminergic deficits in scans, suggesting different disease mechanisms. Parkinsonism includes typical PD motor symptoms. In these cohorts, specific miRNAs were identified as potential biomarkers, reflecting the molecular changes associated with each subtype (Figure 1).^20^

MicroRNAs (miRNAs) are emerging as significant biomarkers in PD due to their stability in human fluids, and their role in gene regulation.^21^^,^^22^ However, this area of research faces challenges due to the non-specific nature of some miRNAs, which are also implicated in other diseases. This limits their specificity for PD diagnosis. Further, the expression levels of key miRNAs can vary based on factors such as drug interactions, necessitating careful consideration in experimental designs.^23^ These challenges highlight the need for robust and reliable resources such as the PD map, which provides a comprehensive and expert-reviewed knowledge base resource.

The PD map serves as a valuable knowledge repository, providing high-quality, disease-specific information crucial for computational modeling. The PD map can be translated into a dynamic Boolean model, allowing for dynamic simulation and predictions. Boolean models offer a simple approach to represent complex disease mechanisms. The biomolecules in a disease mechanism can be represented as model components and their interactions are described by Boolean functions. Boolean models are advantageous as they do not require detailed kinetic information, making them suitable for large-scale data analysis and hypothesis testing. PBNs extend the basic Boolean model by incorporating stochastic elements, which more accurately reflect the inherent variability and dynamic nature of biological systems.^10^^,^^24^ In this context, we used pyMaBoss, in which a biological system is depicted as a network of interconnected Boolean variables, each of which represents the state of a biomolecule (e.g., present or absent, active or inactive). Boolean rules, which determine how one variable’s state can influence another’s, define the interactions between these variables. pyMaBoss employed the Monte Carlo algorithm operates by randomly sampling possible system states at each time step. This sampling is based on the probabilities of each state given the system’s current state and the rules dictating variable interactions. Through multiple time step simulations, PyMaBoSS can estimate the probability of each state at every point in time to simulate the dynamics of a model.^25^ To this end, Boolean modeling has significant applications in clinical and translitional medical research for a range of purposes. Simulations of complex biological systems have enabled the prediction of pathway endpoint activities, drug targets, and cellular crosstalks. Identifying attractors has provided an understanding of phenotype activities as they represent steady states of components. Furthermore, attractor comparisons before and after perturbations can shed light on how *in vivo* systems maintain their homeostasis.^26^^,^^27^^,^^28^^,^^29^^,^^30^^,^^31^^,^^32^^,^^33^

## Results

To study complex molecular mechanisms of PD, we analyzed PPMI cohort data to focus on significant pathways associated with PD pathogenesis. We used this information to construct and parametrize Boolean models. Once the models were analyzed, we compiled, interpreted, and validated our findings. The validation process involved the consistency of the model results with the existing literature to ensure their alignment with the known biological behavior and experimental data. We identified pathways specific for mitochondrial dysfunction and insulin resistance, common across various PD subgroups and T2DM comorbidities, highlighting their critical roles in disease pathogenesis.

### Analysis of expression profiles for pathway modeling

#### MicroRNAs data analysis for Parkinson’s disease subgroups

We calculated the differential expression of miRNAs and their standardized effect sizes and transformed them into probabilities using the Common Language Effect Size method. Based on manually curated miRNA databases we identified miRNA targets. The targets were filtered based on the substantia nigra dataset^34^ and compared with those reported in published literature and datasets. We found that most of the significant miRNAs were downregulated in PD and were involved in mitochondrial dysfunction. The miRNAs that did not match with the published validated experiments were not considered in further analysis (Table 1).

**Table 1:** Matched vs. Mismatched PPMI-miRNAs in Literature

	miRNA	Regulation	Sample	Method	References
Matched expressions	hsa-miR-96-5p	Up	Peripheral blood	RT-qPCR (TaqMan)	Alieva et al.^35^
	hsa-miR-26a-5p	Up	Peripheral blood	RT-qPCR (SYBR Green)	Botta-Orfila et al.^36^
	hsa-miR-424-5p	Up	Peripheral blood	Microarray	Botta-Orfila et al.^36^
	hsa-miR-9-3p	Up	Peripheral blood	RT-qPCR (TaqMan)	Botta-Orfila et al.^36^
	hsa-miR-454-3p	Up	Peripheral blood	RT-qPCR (TaqMan)	Cardo et al.^37^
	hsa-miR-15b-5p	Up	Peripheral blood	RT-qPCR (TaqMan)	Cardo et al.^37^
	hsa-miR-671-5p	Up	Peripheral blood	Microarray	Chi et al.^38^
	hsa-miR-93-5p	Up	Prefrontal cortex	Illumina’s HiSeq 2000	Chi et al.^38^
	hsa-miR-195-5p	Up	Peripheral blood	RT-qPCR (TaqMan)	Ding et al.^39^
	hsa-miR-20a-5p	Up	Peripheral blood	Microarray	Ding et al.^39^
	hsa-miR-16-5p	Up	Prefrontal cortex	Illumina’s HiSeq 2000	Ding et al.^39^
	hsa-miR-132-3p	Up	Peripheral blood	RT-qPCR (TaqMan)	Hoss et al.^40^
	hsa-miR-196b-5p	Down	Peripheral blood	RT-qPCR (TaqMan)	Hoss et al.^40^
	hsa-miR-92b-3p	Down	Peripheral blood	Microarray	Hoss et al. ^40^
	hsa-miR-19a-3p	Down	Mid-brain	Microarray	Hoss et al.^40^
	hsa-miR-19a-3p	Down	Peripheral blood	Microarray	Hoss et al.^40^
	hsa-miR-92a-3p	Down	Peripheral blood	RT-qPCR (TaqMan)	Hoss et al.^40^
	hsa-miR-133b	Down	Mid-brain	RT-qPCR (TaqMan)	Hoss et al.^40^
	hsa-miR-15b-5p	Down	Peripheral blood	RT-qPCR (TaqMan)	Hoss et al.^40^
	hsa-miR-7-5p	Down	Peripheral blood	RT-qPCR (TaqMan)	Alieva et al.^35^
	hsa-miR-15a-5p	Down	Peripheral blood	Microarray	Khoo et al.^41^
	hsa-miR-19b-3p	Down	Peripheral blood	RT-qPCR (TaqMan)	Martins et al.^42^
	hsa-miR-139-5p	Down	Peripheral blood	RT-qPCR (TaqMan)	Martins et al.^42^
	hsa-miR-450b-5p	Down	Peripheral blood	RT-qPCR (TaqMan)	Martins et al.^42^
	hsa-miR-212-3p	Down	Prefrontal cortex	Illumina’s HiSeq 2000	Martins et al.^42^
	hsa-miR-22-3p	Down	Peripheral blood	RT-qPCR (TaqMan)	Martins et al.^42^
	hsa-miR-26a-5p	Down	Peripheral blood	RT-qPCR (TaqMan)	Martins et al.^42^
	hsa-miR-16-2-3p	Down	Prefrontal cortex	Illumina’s HiSeq 2000	Martins et al.^42^
	hsa-miR-16-2-3p	Down	Peripheral blood	RT-qPCR (TaqMan)	Margis et al.^43^
	hsa-miR-30b-5p	Down	Peripheral blood	RT-qPCR (TaqMan)	Margis et al.^43^
	hsa-miR-144-3p	Down	Prefrontal cortex	Illumina’s HiSeq 2000	Serafin et al.^44^
	hsa-miR-323a-3p	Down	Peripheral blood	RT-qPCR (TaqMan)	Soreq et al.^45^
	hsa-miR-495-3p	Down	Peripheral blood	RT-qPCR (TaqMan)	Soreq et al.^45^
	hsa-miR-148b-3p	Down	Peripheral blood	RT-qPCR (TaqMan)	Soreq et al.^45^
	hsa-miR-374a-5p	Down	Peripheral blood	RT-qPCR (TaqMan)	Soreq et al.^45^
	hsa-miR-199b-3p	Down	Peripheral blood	RT-qPCR (TaqMan)	Soreq et al.^45^
	hsa-miR-374b-3p	Down	Peripheral blood	RT-qPCR (TaqMan)	Soreq et al.^45^
	hsa-miR-20a-5p	Down	Peripheral blood	Microarray	Soreq et al.^45^
Mismatched expressions	hsa-miR-199b-3p	Up	Peripheral blood	Microarray	Soreq et al.^45^
	hsa-miR-196b-5p	Up	Peripheral blood	RT-qPCR (TaqMan)	Ravanidis et al.^46^
	hsa-miR-221-3p	Up	Peripheral blood	RT-qPCR (TaqMan)	Ravanidis et al.^46^
	hsa-miR-103a-3p	Up	Peripheral blood	RT-qPCR (TaqMan)	Ravanidis et al.^46^
	hsa-miR-320b	Up	Prefrontal cortex	Illumina’s HiSeq 2000	Ravanidis et al.^46^
	hsa-miR-30c-5p	Down	Peripheral blood	RT-qPCR (TaqMan)	Alieva et al.^35^, Ravanidis et al.^46^
	hsa-miR-30a-5p	Down	Peripheral blood	RT-qPCR (SYBR Green)	Margis et al.^43^ and Ravanidis et al.^46^
	hsa-miR-181c-5p	Down	Peripheral blood	RT-qPCR (TaqMan)	Vallelunga et al. ^47^
	hsa-miR-338-5p	Down	Prefrontal cortex	Illumina’s HiSeq 2000	Vallelunga et al. ^48^
	hsa-miR-148b-3p	Down	Prefrontal cortex	Illumina’s HiSeq 2000	Cao et al. ^49^
	hsa-miR-21-5p	Down	Peripheral blood	Microarray	Botta-Orfila et al.^36^, Cao et al.^49^

The table indicate the matched (top) and mismatched (bottom) expressions between the filtered PPMI-miRNAs and the reported miRNAs in literature and datasets. The table includes the miRNA name, the direction of regulation (up or down), the sample type, the method used for measurement, and the reference for the data.

All the miRNAs reported in the literature appear as differentially expressed in our analysis. The majority of the identified miRNAs are dysregulated in all cohorts (see Table 1). The effect size of the filtered miRNAs differs between cohorts (see Limitations of the study).

#### RNAseq data analysis for type 2 diabetes mellitus comorbidity

To identify potential connections between PD and T2DM, we analyzed transcriptomic data from PINK1 and GBA mutations in T2DM organoid models and compared them to a substantia nigra dataset of PD, as described in Modelling-based patient stratification by disease subgroup analysis. The following results were obtained.

(1) Differentially expressed genes in two datasets describing the PINK1 Q456X and GBA N307S mutations in T2DM were identified and common overlaps with the genes in the substantia nigra dataset on the PD Map were determined.^12^ It was found that 81 genes are commonly altered across datasets (see the Supplementary File)
(2) Validated common miRNA-target pairs that were reported in the literature as being involved in both PD and T2DM were identified (Table 2) through enrichment analysis of the differentially expressed genes (DEGs) and a literature search. Among the significant DEGs analyzed, a subset of 20 was found to overlap with the substantia nigra dataset.

**Table 2:** Shared miRNA Expression and Target Genes in PD and T2DM

miRNA	Targets	References	
		PD	T2DM
hsa-miR-423-3p	CDKN1A	da Silva et al.^50^	Blum et al.^51^
hsa-miR-132-3p	MAPK1	da Silva et al.^50^	Zhou et al.^52^ and Mziaut et al.^53^
hsa-mir-15a-5p	RET, PHLPP1	da Silva et al.^50^	Al-Kafaji et al.^54^ and Houshmand-Oeregaard et al.^55^
hsa-mir-29c-3p	PTEN	Bai et al.^56^	Massart et al.^57^
hsa-mir-29a-3p	IGF1	Bai and Goh et al.^56^^,^^58^	Massart.^57^, Dooley et al.^59^
hsa-mir-20a-5p	PTEN, E2F1	da Silva et al.^50^, Chatterjee et al.^60^	Ye et al.^61^, Pheiffer et al.^62^
hsa-mir-22-3p	PTEN	Barbagallo et al.^63^	Senese et al.^64^
hsa-mir-26b-5p	IGF1R, PTEN	Martinez et al.^65^	Liang et al.^66^
hsa-mir-143-3p	IGF1R, AKT1	da Silva et al.^50^	Xihua et al.^67^
hsa-mir-145-5p	IGF1R, IRS1, EIF4E, RPS6KB1	Chen et al.^68^	Cui et al.^69^
hsa-mir-133b	IGF1R, AKT1	Zhang et al.^70^	Y et al.^71^
hsa-mir-34a-5p	E2F1	Rostamian Delavar et al.^72^	Kokkinopoulou et al.^73^
hsa-mir-182-5p	PTEN, GSK3B	Roser et al.^74^	Weale et al.^75^
hsa-mir-148a-3p	IRS1	Martinez et al.^65^	Mononen et al.^76^
hsa-mir-7-5p	SNCA, IGF1R, RS1	da Silva et al.^50^ and Martinez et al.^65^	Wan et al.^77^
hsa-mir-195-5p	RET, INSR	Martinez et al.^65^	Wang et al.^78^
hsa-mir-218-5p	RET	Xing et al.^79^	Yao et al.^80^
hsa-mir-200c-3p	ROCK2	Chatterjee et al.^60^	Satake et al.^81^
hsa-miR-125b-2-3p	IGF1R	Fan et al.^82^	Yu et al.^83^
hsa-mir-18a-5p	PTEN, PHLPP1	Chatterjee et al.^60^	Vasu et al.^84^
hsa-miR-17-5p	PHLPP1, PTEN,E2F1	Xing et al.^79^	Shaker et al.^85^
hsa-mir-96-5p	GSK3B	Dong et al.^86^	Jeong et al.^87^
hsa-mir-21-5p	PTEN, E2F1	Zhao et al.^88^	Mazzeo et al.^89^
hsa-mir-200a-3p	PTEN, MAPK14	Fu et al.^90^	Assmann et al.^91^
hsa-miR-200b-3p	PHLPP1, ROCK2	Fu et al.^90^	Assmann et al.^91^
hsa-miR-200c-3p	ROCK2	Fu et al.^90^	Assmann et al.^91^
hsa-mir-103a-3p	PTEN	da Silva et al.^50^	Assmann et al.^91^
hsa-mir-10a-5p	PTEN	Roser et al.^22^	Zhang et al.^92^
hsa-mir-153-3p	SNCA, PTEN	Roser et al.^22^	Sun et al.^93^
hsa-mir-19b-3p	PTEN	Roser et al.^22^	Akhbari et al.^94^
hsa-mir-155-3p	PTEN	Goh et al.^58^	Tang et al.^95^
hsa-mir-26a-5p	GSK3B, PRKCD, PTEN	Goh et al.^58^	Jiang et al.^96^
hsa-miR-26b-5p	IGF1R, PTEN	Goh et al.^58^	Jiang et al.^96^
hsa-let-7a-5p	E2F1	Goh et al.^58^	Frost et al.^97^
hsa-mir-23a-3p	PTEN	Barbagallo et al.^63^	de Candia et al.^98^
hsa-mir-100-5p	AKT1, IGF1R	Taguchi et al.^99^, Peng et al.^100^	Assmann et al.^101^
hsa-mir-92a-3p	PHLPP1, PTEN	Taguchi et al.^99^	Bhatwadekar et al.^102^

The table indicates the common miRNA expression and target regulation in PD and T2DM. The table lists miRNA, targeted genes, and references for studies that have common altered expression of the miRNA in PD or T2DM.

To identify pathways plausible for Boolean modeling, we performed enrichment analysis for the targets of miRNAs identified in the PPMI cohort. Using the PD map as a pathway repository, this analysis produced significant pathways as listed in Table 3. These pathways were translated into Boolean models (BMs) and subsequently parameterized in accordance with effect size computations (Table 5).

**Table 3:** Input-Output Boolean Models for Dopamine, Wnt-PI3K-AKT, FOXO3, and mTOR-MAPK Pathways

Model	*p* value	Simulation inputs	Simulation outputs
Dopamine transcription	1.42E-14	ADCYAP1, BDNF, EN1, FOXO1, GCH1, PBX1, PRKAA2, RXRA, SESN3, SFPQ, RGS6, NRF1, MAP1B, LMX1A, FOXO3	Mitochondrial biogenesis Dopamine metabolism Neuron survival
\nextWnt-PI3KAKT	2.83E-24	AKT1, E2F1, EIF2AK3, GSK3B, IGF1, IGF1R, IRS1, MAPK1, NEDD4, PRKCD, PTEN, TFDP1, AGO2, EIF4E, IDE, PHLPP1, PPP2CA, PPP2CB, ROCK2, RPS6KB1	TFEB phosphorylated Insulin resistance TFEB SNCA complex TFEB complex Neuron death
\nextFOXO3 activity	1.30E-20	AKT1, BCL2L11, CEBPB, FASLG, GABARAPL1, MAP3K5, MFN2, PPARGC1A,RICTOR, SESN3, SIRT1, SOD2, TXNIP, ATG12, BNIP3, FOXO3, HSPD1, JUN	Response to oxidative stress Fission Fusion Autophagy Mitochondrial biogenesis Apoptosis
\nextmTOR-MAPK signaling	1.39E-22	AKT1, DEPTOR, GSK3B, MAPK1, MTOR, PRKAA1, PRKAA2,RHEB, RICTOR, RRAGD, SIRT1, TSC1, UBE2V1, CAMKK2, DDIT4, DEPDC5, DEPTOR, GSK3B,MAPK1, MAPKAP1, MTOR, PARP1,PHLPP1, PRKAA1, RHEB, RICTOR, RPS6KB1, SIRT1, TSC1, UBE2V1	Glycolysis RHEB lysosome AKT Catabolism Autophagy Mitochondrial biogenesis
\nextPRKN	4.47E-05	ATXN3, BAG4, FBXW7, GABARAPL1, TIMM17A, ULK1, VPS13C	Apoptosis Mitophagy PRKN ubiquitinated PINK1 accumulation

This table presents the Boolean models, with the inputs and outputs for each model listed. The pathways included are the Dopamine transcription pathway, the Wnt-PI3KAKT pathway, the FOXO3 activity pathway, and the mTOR-MAPK signaling pathway. The inputs for each pathway consist of various biomolecules, while the outputs represent various cellular processes or biomolecules that are influenced by the inputs.

### Construction of models of Parkinson’s disease pathways

We constructed Boolean models based on the PPMI cohort-specific dataset and the PD map. This helped us choose the significant diagrams for further analysis and modeling. The criteria for selecting diagrams for subsequent modeling and stratification were based on the pathway enrichment analysis of the PPMI dataset using the PD map. The enriched pathways included dopamine transcription pathways, PI3k/AKT signaling, FOXO3 activity, mTOR-MAPK signaling, and PRKN mitophagy. These pathways emphasize that the consequences of their dysregulation are largely dependent on the characteristics of the disease subgroups. To ensure comprehensive coverage of all disease subtypes under consideration, we incorporated all miRNA targets from the PPMI dataset into the enrichment analysis.

The pathways identified via the enrichment analysis were exported from the PD map in CellDesigner SBML format and translated into SBML-qual files by the CaSQ tool. These SBML-qual files were first verified for correctness and completeness. To this end, we performed structural and dynamical analysis. Structural verification involves the assessment of interactions among the biomolecules of the model. The assessment examined the interactions between the biomolecules, focusing on the nature of the interactions. The examination was achieved by using the SIGNOR database^103^ to verify the type of the interactions-whether inhibitory or stimulatory. Additionally, the assessment involved the identification of the directionality of these interactions using the same database.

Dynamic verification examined the model’s dynamic behavior over iteration steps, evaluating the model response against single perturbations. We altered the state of single nodes to observe the effects on the model behavior. We validated the reliability of our BMs behavior by comparing their behavior with actual data. Through simulations, we assessed the capacity of BMs to mimic known perturbations and their reliability in modeling corresponding biological processes. The simulated behavior of these pathways matched the expected behavior according to published literature (See Table 4). The simulated pathways’ responses were compared to expected biological outcomes and the coherence of dynamic patterns observed in the literature. We conducted the comparison by examining each model’s simulated responses that reflect the expected biological behaviors. The metrics to decide the the model behaved correctly were qualitative measures. We aligned the ON/OFF state transitions of each biomolecule in the simulation with the corresponding activation/inhibition behavior described in the literature. We considered the model accurate when an ON state corresponded to activation behavior and an OFF state corresponded to inhibition, as per established biological findings.

**Table 4:** Simulated and expected outcomes of boolean models for key biological pathways

Pathway	Dimension					
	Nodes	Edges	Target node	State	Simulated behavior	Expected behavior
PGC1 alpha	69	109	PPARGC1A	ON	Mitochondrial biogenesis	Match (Da Cruz et al.^104^)
			SIRT1	ON	Mitochondrial biosynthesis	Match(Stoyas et al.^105^)
Dopamine transcription	167	196	NR4A2	OFF	Dopamine release	Match (Zhang et al.^106^)
Wnt/PI3K	391	436	LRRK2	ON	Autophagy activation	Match(Bravo-San Pedro et al.^107^)
						Mismatch (Albanese et al.^108^)
			Wnt	ON	Increase auto-phagy	Match (Lorzadeh et al.^109^)
			DDIT3	ON	increase BCL2L11/BBC3i	Match (Zhu et al.^110^)
			GSK3B	OFF	Autophagy activation	Match (Hermida et al.^111^)
			TFEB	ON	Autophagy activation	Match (Zhuang et al.^112^)
			PHLPP	OFF	Autophagy deactivation	Match (Li et al.^113^)
			RPS6KB1	OFF	Autophagy deactivation	Match (Li et al.^113^)
			4EBP2	ON	Autophagy activation	Match (Silva et al.^114^)
FOXO3 activity	65	86	FOXO3	ON	Autophagy activation	Match (Fasano et al.^115^)
					BNIP3 activation	Match (Fasano et al.^115^)
TCA cycle	137	160	AKDHC	OFF	acetyl coA-ATP-NADH	Match (Kim et al.^116^)
			Oxoglutarate	OFF	acetyl coA-ATP-NADH	Match (Kim et al.^116^)
			IDH	OFF	Acetyl coA-ATP-oxoglutarate	Match (Kim et al.^116^)
			SIRT3	OFF	Acetyl coA-ATP-Iron	Match (Kim et al.^116^)

The table compares the simulated behavior of several Boolean models to expected behavior based on published literature. The table includes information on the pathways, the number of nodes and edges in each network, the target node, the state of the target node (ON or OFF), and the simulated and expected behavior for each pathway.

### Model parameterization using cohort data

Using the miRNA expression data of the PPMI dataset we determined the probabilities of the initial states of BMs, based on the effect size and statistical correlations of each subgroup with the control (PD clinical). We used these calculated probabilities in BM models constructed from the PD map to run simulations in pyMaBoSS. With these simulations of BM, we explored the likelihoods associated with reaching levels of specific model outputs, listed in Table 3. With this, we evaluated the impact of molecular changes on the states of cellular phenotypes.^29^^,^^117^^,^^118^

The illustration in Table 5 shows the computed miRNA effect sizes specific to the SWEDD, prodromal, and parkinsonism subtypes, along with associated targets. When this tailored model is simulated using pyMaBoSS, it yields output readouts for the three phenotypes (see Limitations of the study).

**Table 5:** PRKN-mitophagy Model parameters and effect sizes in SWEDD, prodromal, and parkinsonism

Cohort	Target.Score	miRNA	Gene.ID	Gene.Symbol	Transcript.Accession	CL-effectsize
SWEDD	95	hsa-miR-96-5p	4287	ATXN3	NM_001164776	0.966761
	97	hsa-miR-26a-5p	9530	BAG4	NM_001204878	0.712208
	97	hsa-miR-424-5p	55294	FBXW7	NM_033632	0.852718
	95	hsa-miR-15b-5p	23710	GABARAPL1	NM_031412	0.92637
	95	hsa-miR-3121-3p	10440	TIMM17A	NM_006335	0.981296
	96	hsa-miR-26a-5p	8408	ULK1	NM_003565	0.712208
Prodromal	95	hsa-miR-96-5p	4287	ATXN3	NM_001164776	0.956198
	97	hsa-miR-26a-5p	9530	BAG4	NM_001204878	0.951438
	97	hsa-miR-15b-5p	55294	FBXW7	NM_033632	0.960511
	95	hsa-miR-15b-5p	23710	GABARAPL1	NM_031412	0.960511
	95	hsa-miR-3121-3p	10440	TIMM17A	NM_006335	0.574494
	96	hsa-miR-26a-5p	8408	ULK1	NM_003565	0.951438
Parkinsonism	96	hsa-miR-1271-5p	4287	ATXN3	NM_001164776	0.976557
	97	hsa-miR-26b-5p	9530	BAG4	NM_001204878	0.896051
	100	hsa-miR-32-5p	55294	FBXW7	NM_033632	0.914474
	96	hsa-miR-195-5p	23710	GABARAPL1	NM_031412	0.938137
	99	hsa-miR-421	9868	TOMM70	NM_014820	0.942465
	96	hsa-miR-26b-5p	8408	ULK1	NM_003565	0.896051
	98	hsa-miR-223-5p	54832	VPS13C	NM_018080	0.964472

This table presents PRKN-mitophagy Boolean model parameters within three subgroups SWEDD, prodromal, parkinsonism with common language (Cl) effect sizes and targets.

In the simulated graphs shown in (Limitations of the study), we calculated their self-similarity and compared them pairwise to other simulated conditions, as described in Methods (Section 3.5.3). Through the process of comparing graphs pairwise, we can identify which aspects of the system remain consistent across different simulations and which ones vary. This helps in pinpointing recurring or unique patterns within the simulation, which are key to understanding the model behavior. For instance, if two simulated endpoints of a biological system reveal similar patterns despite changes in certain parameters. The observed differences between the simulations can highlight the impact of specific parameters on model endpoints in different groups.

The selected models were additionally parameterized to investigate the comorbidity of PD and T2DM. Utilizing the DEGs identified from the T2DM datasets, perturbations were introduced within these models.

DEGs identified with high expression levels were set in the models as permanently activated (simulated overexpression), and downregulated DEGs were set as permanently inactive (simulated knockout). As illustrated in Table 6, the model of PRKN pathway simulating PD-T2DM comorbidity uses two sets of parameters: i) miRNA-based and ii) specific to T2DM. During simulation, these sets of parameters are used together. The miRNA-based parameters represent characteristics of a given cohort, and the T2DM-specific parameters encode the impact of T2DM on the evolution of the PD cohort.

**Table 6:** Example of PARKIN Pathway Parameterization with miRNA and T2DM-Specific Inputs

Cohort	Cohorts -based targets	CL-effectsize	T2DM-specific targets (Knockouts)
SWEDD	ATXN3	0.966761	SNCA
	BAG4	0.712208	BCL2
	FBXW7	0.852718	BNIP3
	GABARAPL1	0.92637	
	TIMM17A	0.981296	
	ULK1	0.712208	
Prodromal	ATXN3	0.956198	
	BAG4	0.951438	
	FBXW7	0.960511	
	GABARAPL1	0.960511	
	TIMM17A	0.574494	
	ULK1	0.951438	
Parkinsonism	ATXN3	0.976557	
	BAG4	0.896051	
	FBXW7	0.914474	
	GABARAPL1	0.938137	
	TOMM70	0.942465	
	ULK1	0.896051	
	VPS13C	0.964472	

The table shows an example of parameterisation for PARKIN pathway, including two sets of parameters (miRNA based and T2DM specific which are combined) during the simulation.

### Cohort specific simulation results

Following the parameterization, the selected BMs were analyzed in two aspects.

(1) Investigation of molecular mechanisms in disease cohorts: parkinsonism, SWEDD, and prodromal
(2) Effect of T2DM comorbidity in these cohorts

For each of the selected models (Table 3), two sets of results were obtained: cohort-specific and comorbidity-specific results.

#### Dopamine transcription

In “Dopamine transcription” BM, during early simulation phases SWEDD and prodromal groups exhibited similar activation levels in mitochondrial genesis and dopamine metabolism. Conversely, the parkinsonism group manifested elevated activation levels for these biological processes in comparison to the other two groups. Noteworthy variances are also evident in neuron survival across all groups, marked by diminished activity within the SWEDD and prodromal groups (Table 7).

**Table 7:** DTW scores in dopamine transcription boolean Model across prodromal, SWEDD, and parkinsonism stages

Stage	Conditions	Cohort miRNA			T2DM transcriptomics		
		Prodromal	SWEDD	Parkinsonism	Prodromal	SWEDD	Parkinsonism
Early	Mitochondrial biogenesis	0.6771	0.6512	0.3853	0.3170	0.3185	0.1937
	Dopamine metabolism	0.4592	0.4565	0.3856	0.1359	0.1322	0.1238
	Neuron survival	0.3743	0.4894	0.3818	0.3413	0.3274	0.0668
Mid	Mitochondrial biogenesis	0.1693	0.1623	0.0493	0.8890	0.8927	0.0456
	Dopamine metabolism	0.0973	0.0680	0.0496	0.0935	0.2073	0.0436
	Neuron survival	0.0761	0.0906	0.0674	0.9226	0.8951	0.0268
Late	Mitochondrial biogenesis	0.0311	0.0482	0.0002	0.9676	0.9698	0.0023
	Dopamine metabolism	0.0132	0.0135	0.0098	0.0364	0.0765	0.0198
	Neuron survival	0.0110	0.0210	0.0295	0.0364	0.0765	0.0198

The table presents the DTW scores in dopamine transcription BM for three different simulation stages of prodromal, SWEDD and parkinsonism. The scores are based on three different disease conditions: mitochondrial biogenesis, dopamine metabolism, and neuron survival.

In the mid and late phases of simulations, we observed a general increase in activation levels for the aforementioned processes across all cohorts. An exception is the T2DM profile, where the activation levels for both mitochondrial functionality and “neuron survival” remain constant. During these middle and late phases, distinct disparities in mitochondrial genesis arise between the SWEDD or prodromal-associated T2DM and other groups (Table 7). Within the parkinsonism-T2DM cohort, the activation intensities for “mitochondrial biogenesis” and “neuron survival” consistently rank lower compared to other groups across all simulation stages. These observations suggest a possible relationship between diabetes and reduced mitochondrial production and neuron survival, especially in cases of parkinsonism-T2DM.

#### Wnt-PI3K/AKT signaling

During the initial stages of simulations, a significant difference in insulin resistance emerges among the prodromal group and the SWEDD and parkinsonism groups. Both the SWEDD and parkinsonism groups exhibit similar levels of insulin resistance, in contrast to the prodromal group. The SWEDD and prodromal groups have similar levels of insulin resistance as a consequence of T2DM. The probability of the TFEB complex activation (the inactive form) is increased, whereas the active forms of TFEB (including phosphorylated TFEB and TFEB SNCA) exhibit a decreased probability of their activation levels (Table 8).

**Table 8:** DTW scores in Wnt-PI3K/AKT boolean Model across prodromal, SWEDD, and parkinsonism stages

Stage	Conditions	Cohort miRNA			T2DM transcriptomics		
		Prodromal	SWEDD	Parkinsonism	Prodromal	SWEDD	Parkinsonism
Early	TFEB phosphorylated	0.9503	0.9215	0.9216	0.9032	0.9053	0.8998
	Insulin resistence	0.6341	0.3364	0.3310	0.1111	0.1118	0.0190
	TFEB SNCA complex	0.9622	0.9422	0.9422	0.8964	0.9215	0.9088
	TFEB complex	0.3624	0.3572	0.3580	0.05755	0.0575	0.0125
	Neuron death	0.3090	0.2714	0.2725	0	0	0.0366
Mid	TFEB phosphorylated	0.9503	0.9215	0.9216	0.8847	0.8906	0.8758
	Insulin resistence	0.6341	0.3364	0.3310	0.0213	0.0203	0.0098
	TFEB SNCA complex	0.9622	0.9422	0.9422	0.9080	0.9065	0.9078
	TFEB complex	0.3624	0.3572	0.3580	0.0007	0.0006	0.0006
	Neuron death	0.3090	0.2714	0.2725	0	0	0.0048
Late	TFEB phosphorylated	0.9298	0.9276	0.9243	0.8997	0.9009	0.8991
	Insulin resistence	0.0241	0.0222	0.0212	0.0213	0.0203	0.0098
	TFEB SNCA complex	0.9324	0.9279	0.9208	0.9066	0.8914	0.8931
	TFEB complex	0.0296	0.0098	0.0097	0	0	0
	Neuron death	0.0192	0.0005	0.0009	0	0	0

The table presents the DTW scores in Wnt-PI3K/AKT BM for three different simulation stages of prodromal, SWEDD and parkinsonism. The scores are based on different disease conditions: TFEB phosphorylated, Insulin resistence, TFEB complex and Neuron death.

#### FOXO3 activity

During the early, mid, and late stages of the simulation, in the prodromal group, we observed increased activation of the Fission Fusion endpoint when compared to other groups. The prodromal-T2DM group had an increased activation of autophagy and oxidative stress endpoints than other groups during the three stages of the simulation. The prodromal and SWEDD groups both show a higher level of oxidative stress endpoint activation compared to the parkinsonism group. At the mid-stage of the simulation, the activation of fission and fusion within SWEDD and parkinsonism groups were similar. Furthermore, parkinsonism group displayed increased activation of autophagy relative to other groups. Also, the activation of apoptosis was similar between the prodromal and SWEDD groups. In the SWEDD group, the activation of autophagy and apoptosis increased in the late stage when compared to the prodromal and parkinsonism groups (Table 9).

**Table 9:** DTW scores in FOXO3 boolean Model across prodromal, SWEDD, and parkinsonism stages

Stage	Conditions	Cohort miRNA			T2DM transcriptomics		
		Prodromal	SWEDD	Parkinsonism	Prodromal	SWEDD	Parkinsonism
Early	Response to oxidative stress	0.6331	0.6220	0.6949	0.4473	0.4850	0.5954
	Fission Fusion	0.6361	0.8529	0.8111	0.4676	0.6192	0.6858
	Autophagy	0.5561	0.6491	0.5514	0.3789	0.3962	0.4145
	Mitochondrial biogenesis	0.5622	0.5419	0.5824	0.2045	0.1843	0.1500
	Apoptosis	0.7547	0.7707	0.5508	0.3947	0.4657	0.3255
Mid	Response to oxidative stress	0.5138	0.4952	0.5708	0.5133	0.4938	0.6417
	Fission Fusion	0.5413	0.6091	0.6015	0.5188	0.7658	0.6431
	Autophagy	0.5096	0.5418	0.4814	0.5195	0.4671	0.5028
	Mitochondrial biogenesis	0.1257	0.1465	0.1633	0.0744	0.1251	0.0777
	Apoptosis	0.4666	0.4640	0.3315	0.4231	0.4450	0.4302
Late	Response to oxidative stress	0.5099	0.4577	0.5485	0.5325	0.4703	0.6460
	Fission Fusion	0.5080	0.5133	0.5469	0.5528	0.6010	0.6156
	Autophagy	0.5399	0.4978	0.5367	0.5323	0.4878	0.5567
	Mitochondrial biogenesis	0.0208	0.0372	0.0712	0.0197	0.0436	0.0392
	Apoptosis	0.3177	0.2623	0.2752	0.2664	0.2940	0.3221

The table presents the DTW scores in FOXO3 BM for three different simulation stages of prodromal, SWEDD and parkinsonism. The scores are based on five different disease conditions: Response to oxidative stress, Fission Fusion, Autophagy, Mitochondrial biogenesis and Apoptosis.

During both the mid and late stages, the SWEDD group had increased activation of oxidative stress endpoint compared to the prodromal and parkinsonism groups. The SWEDD-T2DM group also showed an increased probability of activation in oxidative stress and mitochondrial biogenesis endpoints. The prodromal group, showed increased activation of the mitochondrial biogenesis endpoint. The mitochondrial biogenesis endpoint within the parkinsonism group was decreased by T2DM condition compared to other cohorts (Table 9).

#### mTOR-MAPK signaling

During the early stage of the simulation, pronounced differences emerged in the activity levels of glycolysis and catabolism across all groups. Among these, in the SWEDD group, we observed a change in glycolysis activation earlier and its elevated activity continued into the late stage. Conversely, the SWEDD-T2DM group showed reduced glycolysis endpoint levels at every stage of T2DM related cohorts, with an accompanying rise in catabolism and diminishing activation levels of glycolysis (Table 10). In the parkinsonism group, catabolic activity is more pronounced during the early and middle stages in comparison to the SWEDD and prodromal groups. Although the activation of glycolysis is increased, the SWEDD group demonstrated increased levels of catabolism during the mid to late stages (Table 10). In the parkinsonism-T2DM group, we observed increased activation of glycolytic activity endpoint compared to other groups with T2DM in all simulation stages (Table 10).

**Table 10:** DTW Scores in mTOR Boolean Model Across Prodromal, SWEDD, and Parkinsonism Stages

Stage	Conditions	Cohort miRNA			T2DM transcriptomics		
		Prodromal	SWEDD	Parkinsonism	Prodromal	SWEDD	Parkinsonism
Early	Glycolysis	0.8151	0.7664	0.9058	0.3040	0.3971	0.1740
	RHEB lysosome	0.6653	0.3861	0.6113	0.1382	0.0098	0.0286
	AKT	0.4171	0.3893	0.4172	0.0630	0.0122	0.0081
	Catabolism	0.8087	0.8350	0.7342	0.3553	0.4528	0.1848
Mid	Glycolysis	0.3077	0.3461	0.5149	0.3365	0.3690	0.2926
	RHEB lysosome	0.2368	0.0937	0.2166	0.0744	0.0098	0.0048
	AKT	0.1115	0.0962	0.1930	0.0555	0.0098	0.0065
	Catabolism	0.3688	0.3193	0.2579	0.3458	0.2822	0.3093
Late	Glycolysis	0.1493	0.1397	0.1780	0.1666	0.1799	0.1458
	RHEB lysosome	0.1227	0.0446	0.0643	0.0492	0.0002	0.00003
	AKT	0.0427	0.0296	0.0439	0.0128	0.0001	0.00001
	Catabolism	0.1601	0.1392	0.1398	0.1924	0.1629	0.2083

The table presents the DTW scores in mTOR BM for three different simulation stages of prodromal, SWEDD and parkinsonism. The scores are based on four different disease conditions: Glycolysis, RHEB lysosome, AKT and Autophagy.

#### PRKN mitophagy

At all simulation stages, there was a significant difference in the activation of mitophagy across groups. Within the SWEDD and prodromal groups, mitophagy endpoint activation began earlier during the early simulation stages in comparison to the parkinsonism group. The latter group had an elevated activation of PINK1 accumulation relative to other cohorts. Additionally, the T2DM comorbidity reduced the activation of the mitophagy endpoint specifically within the parkinsonism group (Table 11).

**Table 11:** DTW scores in PRKN Mitophagy boolean Model across prodromal, SWEDD, and parkinsonism stages

Stage	Conditions	Cohort miRNA			T2DM transcriptomics		
		Prodromal	SWEDD	Parkinsonism	Prodromal	SWEDD	Parkinsonism
Early	Mitophagy	0.3303	0.3303	0.4719	0.0537	0.0537	0.0422
	PRKN ubiquitinated	0.6165	0.6165	0.4545	0.2041	0.2041	0.0643
	PINK1 accumulation	0.5952	0.5952	0.5870	0	0	0.1556
Mid	Mitophagy	0.0325	0.0325	0.0820	0.0078	0.0078	0.0335
	PRKN ubiquitinated	0.1918	0.1918	0.1009	0.1065	0.1065	0.1212
	PINK1 accumulation	0.1982	0.1982	0.1314	0	0	0.0658
Late	Mitophagy	0.0002	0.0002	0.0129	0	0	0.0101
	PRKN ubiquitinated	0.0644	0.0644	0.0933	0.0327	0.0327	0.0534
	PINK1 accumulation	0.0747	0.0747	0.0196	0	0	0.0204

The table presents the DTW scores in PRKN mitophagy BM for three different simulation stages of prodromal, SWEDD and parkinsonism. The scores are based on three different disease conditions: Mitophagy, PRKN ubiquitinated, and PINK1 accumulation.

Our Boolean modeling approach enables a detailed examination of mechanistic trajectories within reconstructed pathways by capturing the dynamic interplay of molecular interactions. For example, In our Boolean models, trajectory changes in the dopamine transcription and PI3K/AKT signaling pathways illustrate the integration of disease mechanisms. For example, in the dopamine transcription pathway, dysregulation of NR4A2 and GCH1 leads to impaired dopamine synthesis. This is reflected in the model’s trajectories, showing a decrease in the probability of active dopamine synthesis states, thus illustrating the progression from a healthy to a diseased state in PD. Similarly, in the PI3K/AKT signaling pathway, the model shows how changes in the activation of AKT1, GSK3B, and PTEN disrupt cell survival and metabolism. The trajectories indicate a gradual increase in dysregulated states, correlating with increased neuronal death and metabolic dysfunction. This method provides a deeper understanding of disease mechanisms beyond static analyses. Additionally, we validated our results using literature evidence, including GSEA studies (e.g.,^34^), which confirm the reliability of our mechanistic insights. This comparison shows that, while GSEA identifies significant pathways, our Boolean modeling captures the dynamic regulatory mechanisms and sequential molecular events leading to dysregulation.

#### Similar characteristics in the disease subgroups

Next, we compared the simulated model components among disease subgroups using the Dynamic Time Warping (DTW) algorithm (see Methods). A decreased DTW score between simulated model componets across different subgroups signifies a higher similarity in how these components unfold over time. We used the Pearson correlation coefficient to analyze DTW scores of model components across these subgroups. A Pearson correlation coefficient close to 1 indicated a strong correlation. By examining the DTW scores that are highly correlated (close to 1), we can determine a strong similarity in the temporal patterns of the analyzed subgroups see (Table 12).

**Table 12:** Highly correlated disease conditions across subgroups at various simulation stages

Disease subgroups	Conditions in stage		
	Early	Mid	Late
Prodromal+T2DM, SWEDD+T2DM	Insuline resistence	Insuline resistence	Insuline resistence
	Mitophagy	Mitophagy	Mitophagy
	Mitochondrial biogenesis	Apoptosis	Mitochondrial biogenesis
SWEDD+Parkinsonism	Insuline resistence	Fission fusion	Catabolism
Prodromal+SWEDD	Mitophagy	–	Mitophagy
			Dopamine metabolism
Prodromal+Parkinsonism	Autophagy	–	Autophagy
	Neuron survival		
T2DM (all cohorts)	Dopamine metabolism	–	Neuron death

The table presents the highly correlated disease conditions within disease subgroups (ranging from 98% to 100%) at various stages of simulation using the Pearson correlation coefficient.

All results presented below demonstrate consistent patterns across the simulation stages, highlighting similarities in disease groups. The result is structured into two main points: one examining the collective trends across all disease groups, and the other focusing on pairwise comparisons between specific groups to identify the pairwise similarities.

In all disease groups, we observed an increase in the “mitochondrial dysfunction” endpoint in the early stage of the disease. Further, other cellular endpoints such as “apoptosis” and “dopamine metabolism” are increased. As the simulation advanced to its later stages, the effect of Type 2 Diabetes Mellitus (T2DM) was observed across all groups. As a result of T2DM, the endpoints of “neuron survival” and “dopamine metabolism” were decreased.

As a result of comparing pairs of disease groups, similar patterns among the groups were observed as follows: i) For the prodromal and SWEDD groups, the early stages were characterized by an increase in “mitochondrial biogenesis” endpoints influenced by T2DM and signs of insulin resistance. Additionally, We observed an increase in “neuronal survival” and “Autophagy” endpoints. In the late stages, these groups continued to show an increase in the “mitochondrial biogenesis” endpoint associated with T2DM. Moreover, we observed an increase in the “mitophagy activity” endpoint and disruptions in the “dopamine metabolism” endpoint. ii) For the SWEDD and parkinsonism groups, the early stages demonstrated elevated levels of “insulin resistance” endpoint. In the mid-stage, we observed an increase in the “fission metabolism” endpoint. In the late stage, an increase in “catabolism endpoints” was observed.

## Discussion

Molecular and cellular pathology in Parkinson’s disease (PD) is complex and manifests in a range of symptoms and progression patterns, including comorbidities such as type 2 diabetes mellitus (T2DM).^119^ Understanding these multifaceted interactions is crucial for precise diagnostic or therapeutic approaches. For this purpose, we need systems biology approaches to investigate complex interactions between multiple molecular factors to better understand the disease mechanisms.

### Knowledge integration

Given the variability of genetics, molecular biology, and clinical symptoms in PD, large and well-characterized cohort data is essential for identifying underlying patterns and mechanisms of the disease. For this reason, a dataset of miRNAs sequenced from the whole blood of patients with PD was obtained from different disease subtypes from the PPMI study.^20^ MiRNAs are considered a promising biomarker for both diagnosis and prognosis of disease because of their high stability in human fluids.^21^^,^^22^ However, they often have a broad profile of activity, so in this work we considered miRNAs to have validated interactions with genes in brain tissue. We further selected only miRNAs having stable correlations with target gene expression, using curated data resources and published results related to PD, including an expression profile of PD substantia nigra^34^ (see Table 1 for specific references). Most of these filtered, significant miRNAs showed downregulation in PD and were involved in mitochondrial dysfunction. Moreover, to study a potential effect of T2DM comorbidity, we analyzed transcriptomic profiles of PD-related brain organoids under insulin overexposure. Differentially Expressed Genes (DEGs) from this dataset were filtered to match the targets of cohort-specific miRNAs calculated earlier (Table 2). The common DEGs were involved in dopamine related pathways such as dopamine transcription and dopamine metabolism. This involvement suggests that insulin resistance can disrupt the normal functioning of dopamine, a key neurotransmitter in the brain. This disruption leads to impaired dopamine signaling, which is a key aspect of PD pathology. This highlights how insulin resistance contributes to the progression of PD.

Using the list of key genes compiled as above, we identified a list of pathways to construct our models. The pathways were selected from the Parkinson’s disease map (pdmap.uni.lu).^9^ The PD map is a dedicated repository of curated pathways focused on the molecular pathophysiology of PD. The PD map incorporates detailed knowledge of PD-related molecular interactions, particularly within brain tissues. This allows us to simulate and understand disease mechanisms with greater specificity. While miRNA measures from blood reflect systemic changes, they might not capture all the localized molecular activities within the brain. By integrating detailed data from the PD map, we bridge this gap and explore mechanisms that might be underrepresented or absent in blood-based measures. While this approach offers significant insights, it also presents opportunities for further enhancement. Validating the identified mechanisms using multi-omic approaches that integrate both brain and blood samples, along with mRNA and protein measures from the PPMI cohort, can ensure more robust findings. In the future, we aim to extend our results by integrating these findings with mRNA and protein data, thus providing a deeper insight into the molecular underpinnings of PPMI cohorts.

The contents of the PD map are encoded in SBML-compliant format, allowing the construction of computational models. Thus, based on selected pathways, probabilistic BMs were created, and validated for completeness and correctness, and their initial states were chosen based on the calculated miRNA effect sizes of the PPMI dataset. Boolean simulations were then performed based on these cohort-specific parameterizations. Further, T2DM data were used to parameterize the models to reflect this comorbidity. For PD-T2DM simulations, upregulated DEGs in the models were set to permanent activation, and downregulated DEGs to permanent inhibition. Such simulation allowed to separately study the effects of T2DM on PD progression.

We validated our model findings against independent datasets and studies. (see Table 4). We also conducted sensitivity analyses to test our models against single perturbations (knockdown). This shows that our models can accurately reflect biological behavior independently from PPMI-derived data.

### Modelling-based patient stratification by disease subgroup analysis

The selected models were parameterized with cohort-based data and simulated to stratify different molecular mechanisms during disease progression. Simulations of these models represent stages in disease progression. This allowed us to compare molecular activity across the PD subtypes.

#### Specific characteristics in each cohort

Prodromal cohorts exhibit molecular dysregulations that lead to higher probabilities of PD-related motor signs compared to other cohorts. These molecular dysregulations are related to impaired neuronal autophagy.^120^ Further, the models show that in the prodromal cohort, mitochondrial turnover is more frequent (“Fission and Fusion” output) with higher probabilities than other cohorts in the early stages of simulation, and this pattern continues with a higher probability in the mid and late stages of simulation (Table 9). This finding is consistent with previous research suggesting that mitochondrial abnormalities may occur early in the course of PD^121^^,^^122^

In the SWEDD cohort, an interesting aspect is observed inhibition of “Glycolysis and catabolism” output in the mTOR-MAPK signaling model. At the same time the protein RHEB, which has a neuroprotective role, is highly active. This suggests that RHEB may play a role in decreasing catabolic processes and potentially protecting against the development of PD^123^ (Table 10). In the later stages of SWEDD, there is an increase in catabolism, which is the breakdown of molecules to release energy. This increase in catabolism is accompanied by increased glycolysis activity, which is the breakdown of glucose to produce energy. The increased catabolism and glycolysis may indicate stress adaptation. Understanding and targeting these changes could be crucial to control the PD progression.

In the parkinsonism cohort, dopamine transcription and Wnt-PI3K/AKT models show that mitochondrial biogenesis and dopamine transcription change rapidly with lower change points in the mid and late stages simulation. This finding may be due to the fact that the parkinsonism syndrome tends to progress more rapidly than other PD subgroups.^124^

#### Characteristics of prodromal and scans without evidence of dopaminergic deficit cohorts

The models of SWEDD and prodromal cohorts give the similar pattern of activation in dopamine metabolism and mitochondrial biogenesis in the early stages of PD (Table 7). This finding suggests that the early stages of prodromal and SWEDD may refer to a stage at which individuals do not fulfill diagnostic criteria for clinical PD. Moreover, a recent study proposes that patients with SWEDD do not have early PD.^125^

The following findings suggest that even in the absence of dopaminergic neuron deficiency, as seen in SWEDD, oxidative stress may still contribute to neuronal dysfunction and warrants further investigation to fully understand its role in such conditions.^126^ Dopamine transcription and Wnt-PI3K/AKT models suggest that neuronal activity endpoint may be influenced by dopamine metabolism and mitochondrial biogenesis, as these processes are important for energy production and the function of neurons. Specifically, in Wnt-PI3K/AKT, SWEDD, and prodromal conditions show lower levels of neuronal activity compared to parkinsonism in the early stages. It is possible that dopamine metabolism is sustained in the SWEDD and prodromal early stages of simulation for longer periods of time than in the parkinsonism. As a result, dopaminergic neurons may be affected by oxidative stress, leading to a decrease in their activity.^127^ Oxidative stress response may be related to dopamine metabolism and neuronal activity because oxidative stress can damage cells and disrupt normal cellular function, including dopamine metabolism and neuronal activity.^127^ Prodromal and patients with SWEDD exhibit similar oxidative stress responses that are higher than those observed in parkinsonism patients. This may explain the slight differences in dopamine metabolism and lower neuron survival activity in the dopamine transcription pathway observed between these two subtypes and other conditions in the early stages of the simulation.

Glycolysis and catabolism are central processes that are vital for the production of energy in cells. Dysregulation of these processes is observed in a wide range of disease states, including PD. Simulation results in both cohorts show that changes in glycolysis and catabolism occur earlier in SWEDD and prodromal, compared to parkinsonism (Table 10).

Mitophagy is the process of degrading and recycling mitochondria, and changes in this process may affect the function and survival of mitochondria and cells. Dysregulation of mitophagy is implicated in the development of the prodromal and SWEDD (Table 11). In the PRKN mitophagy model, the prodromal and SWEDD cohorts show higher levels of mitophagy activation than those with parkinsonism. This increase is mediated by the protein ULK1, suggesting that the process may be independent of PRKN,^128^ despite higher activation of “PINK1 accumulation” in the simulation for the parkinsonism cohort.

#### Characteristics of T2DM comorbidity

Diabetes-parameterized and cohort-specific models demonstrate a series of differences from the results discussed above. One of the most affected is the Dopamine transcription model. It features significantly lower activation of mitochondrial biogenesis and neuronal survival at the mid and late stages of simulations (Table 7). This is in line with a recent study, linking T2DM to a decline in neuron survival, mitochondrial biogenesis, and dopamine metabolism, where T2DM was associated with oxidative stress and decreased levels of dopamine and its metabolites in the striatum.^119^^,^^129^ Interestingly, in the mid and late stages, the activation of the “dopamine metabolism” endpoint is less decreased in the SWEDD-T2DM cohort than in the early stage of simulation (Table 7).

The Dopamine transcription model parameterized for the parkinsonism-T2DM cohort, in the mid and late stages of mitochondrial biogenesis is less decreased compared to other T2DM cohorts (Table 7). However, in the early stages of the simulations, T2DM comorbidity is found to increase the cellular response to oxidative stress, potentially through the activation of quality control mechanisms such as Autophagy and Fission and Fusion. These processes may increase apoptosis, a form of cell death triggered in response to cellular stress.^126^ Moreover, in the mTOR-MAPK signaling model for parkinsonism-T2DM, we observed higher glycolysis activity and an increase in the inactivated form of RHEB, and the activation of anaerobic glycolysis. This shift toward anaerobic glycolysis is thought to occur as the brain tries to maintain ion homeostasis by providing a limited amount of energy through the breakdown of glucose in the absence of oxygen. However, this process ultimately leads to chemical changes that result in cell death^130^^,^^131^^,^^132^ (Table 10). Finally, the activation of mitophagy is decreased in parkinsonism with T2DM comorbidity, and increased activation of the protein VPS13C, which delays the progression of mitophagy. In support of this, two novel cases are reported of patients who developed dementia and early onset parkinsonism in the absence of VPS13C.^133^

#### Common characteristics across all cohorts

The results show that dysregulation of insulin resistance is observed in all disease subgroups. Insulin resistance is a condition in which the body’s cells do not respond properly to the insulin hormone, leading to high blood sugar levels and an increased risk of diabetes and other health problems.^134^ The BMs suggest that the development of insulin resistance is linked to the activity of the transcription factor TFEB (Table 8). The Boolean models show that the active forms of TFEB tend to have low activity as confirmed in,^135^ while the inactive form of TFEB, found in a complex with the 14-3-3 protein in the cytoplasm, tends to be elevated.^136^ The 14-3-3 proteins are a family of highly expressed brain proteins with neuroprotective effects in multiple PD experimental models.^136^ However, high levels of the inactive form of TFEB suggest a decrease in 14-3-3 proteins, which may increase the aggregation of alpha-synuclein and impair cellular processes, leading to insulin resistance.^134^ The use of antidiabetic drugs has a beneficial role in controlling PD symptoms,^137^^,^^138^^,^^139^ including Metformin, suggested as a neuroprotective drug in the prodromal cohort.^18^ Metformin can reduce alpha-synuclein aggregation and improve cellular processes associated with age-related conditions.^18^^,^^139^ The Boolean model suggests that the dysregulation of TFEB and its regulated genes plays an important role in insulin resistance and controlling mitochondrial function in PD. A recent study shows that abnormalities in TFEB cause a failure of endolysosomal and autophagic pathways.^140^

Our BMs can help to explain hypotheses to understand complex diseases and propose better therapies and diagnostics. The approach provides hypotheses for targeted therapeutic interventions by linking molecular dysregulation patterns to clinical phenotypes. By identifying common cross-talk between different subtypes of the disease, we highlight that PD subtypes may present similar phenotypes but have different underlying causes and regulators. Constructing experimental models representing each subgroup and perturbing the targets predicted by our models can help observe the pathological signatures, supporting the development of personalized treatments.

Boolean modeling approach allows comparing the model attractors to the disease signature and design perturbation experiments that cause a transition of the pathological signature toward a healthy state. As discussed, the results and hypothesis generated by the models were in line with the existing literature findings. The models proposed that the dysregulation of cellular phenotypes (model endpoints) varies among different disease cohorts. The results of the Boolean models can be used to improve similarity-based differential diagnosis in PD. This can be achieved by identifying the common cross-talk between different subtypes of the disease. PD has different subtypes that may be presented with similar phenotypes (endpoints), but have different underlying causes/regulators. As a result, more precise therapeutic strategies need to be developed based on different causes even if they share the same symptoms. This explains that the targets and treatment strategies should be tailored to each disease subgroup. It could be possible to construct experimental models representing each subgroup and perturb the targets predicted by the model to observe the pathological signatures.

### Limitations of the study

Our work faces a number of limitations. First, the stratification of the models relies on miRNAs and in this work, the specificity of miRNAs is validated in independent studies. Indeed, some miRNAs were found to have mismatched expression levels between the PPMI and literature (Table 1), which may stem from various factors such as the presence of other miRNAs, the availability of specific transcription factors, and the overall gene expression profile of the cell.^23^ Also, to simulate the mutation effect of T2DM on PD cohorts, we use a snapshot of dynamic data which is a limited representation of a complex comorbidity of PD. A more comprehensive approach would require analysis of data describing molecular profiles of progression in both disorders over time.

Next, a number of pathway-based models are analyzed separately, while in fact they are interconnected. The integration of pathways may allow a better understanding of the disease progression and therapeutic responses of PD, which requires a broader investigation is necessary.

Another limitation is the granularity of model parameterization, as we only focused on disease subtypes, without considering other factors that may affect the dynamics of the disease, such as gender and age. Finally, our work lacks experimental validation of proposed combinatorial interventions, limiting supporting literature findings.

## Resource availability

### Lead contact

Requests for further information and resources should be directed to and will be fulfilled by the lead contact, Ahmed Abdelmonem Hemedan. (ahmed.hemedan@uni.lu).

### Materials availability

This study did not generate new materials.

### Data and code availability

This paper analyzes existing, publicly available data, as outlined below. The sources of data, code, and other resources are listed in the key resources table, along with their respective accession codes or identifiers.

• **Data:** The Parkinson’s Progression Markers Initiative (PPMI) dataset, used for model parameterization, is available at: https://www.ppmi-info.org/data and accessible based on user agreement. The access identifier is also available in the key resources table. The molecular interaction diagrams related to PD, the PD map, can be accessed at: https://pdmap.uni.lu/.
• **Code:** The code used to generate the results in this study is available on GitLab: https://gitlab.lcsb.uni.lu/lcsb-biocore/publications/hemedan23-boolean-modelling-of-pd. Access identifier details are also provided in the key resources table. This repository also includes Boolean models generated from the PD map.
• **Other items: Software and Tools**
  •The pyMaBoSS Framework is available on GitHub: https://github.com/colomoto/pyMaBoSS.
  •For SBML conversion, the CaSQ tool can be accessed at: https://sysbio.curie.fr/projects/casq/.
  •The MINERVA Platform for PD map analyses, including the GSEA Plugin, is available at: https://minerva.uni.lu.
  •The DESeq2 R Package is available on Bioconductor: https://bioconductor.org/packages/release/bioc/html/DESeq2.html.
  •For SBML format handling, CellDesigner can be found at: https://www.celldesigner.org.
• Access identifiers for these resources are also listed in the key resources table.

## Acknowledgments

This work was supported by funding from the 10.13039/501100007601European Union’s Horizon 2020 research and innovation program under grant agreement No. 733100: SYSCID—A systems medicine approach to chronic inflammatory diseases. The authors acknowledge the Parkinson’s progression markers Initiative (PPMI) for providing the data used in this research. PPMI, a public-private partnership, is funded by the Michael J. Fox Foundation for Parkinson’s Research and funding partners.

## Author contributions

AH: investigation, conceptualization, and writing–original draft. VS: supervision, and writing–review and editing. RS: supervision and writing–review and editing. MO: conceptualization, review and editing, and supervision. All authors contributed to the article and approved the submitted version.

## Declaration of interests

The authors declare no competing interests.

## STAR★Methods

### Key resources table

**Table:** 

REAGENT or RESOURCE	SOURCE	IDENTIFIER
**Deposited data**		

Parkinson’s Progression Markers Initiative-miRNAs dataset	Laboratory of Neuro Imaging (LONI) archive	https://www.ppmi-info.org/data
PD Map	Luxembourg Center for Systems Biomedicine	https://pdmap.uni.lu/

**Software and algorithms**		

pyMaBoSS Framework	GitHub	https://github.com/colomoto/pyMaBoSS
CaSQ Tool for SBML conversion	CaSQ (CellDesigner as SBML-qual)	https://sysbio.curie.fr/projects/casq/
MINERVA Platform (PD map)	MINERVA Platform	https://minerva.uni.lu
DESeq2 R Package	Bioconductor	https://bioconductor.org/packages/release/bioc/html/DESeq2.html
GSEA Plugin for enrichment analysis	MINERVA GSEA Plugin	https://minerva.uni.lu
CellDesigner (SBML formats)	CellDesigner	https://www.celldesigner.org
Boolean modeling framework	Gitlab	https://gitlab.lcsb.uni.lu/lcsb-biocore/publications/hemedan23-boolean-modelling-of-pd

### Method details

To study the mechanisms of PD, we applied a methodology based on high-quality data and specialized knowledge resources see Limitations of the study). Using the PPMI dataset and the PD map repository, we employed probabilistic Boolean Modeling techniques. Key genes targeted by the miRNA identified in the PPMI dataset were used to select relevant pathways in the PD map. Then, these pathways were translated into Boolean models (BMs) and parameterized using the cohort data to represent different PD subtypes. Simulation and analysis of these models provided insight into i) differences in pathway dynamics between studied PD subtypes and ii) potential impact of T2DM comorbidity on these pathways (Figure).

**Figure:**
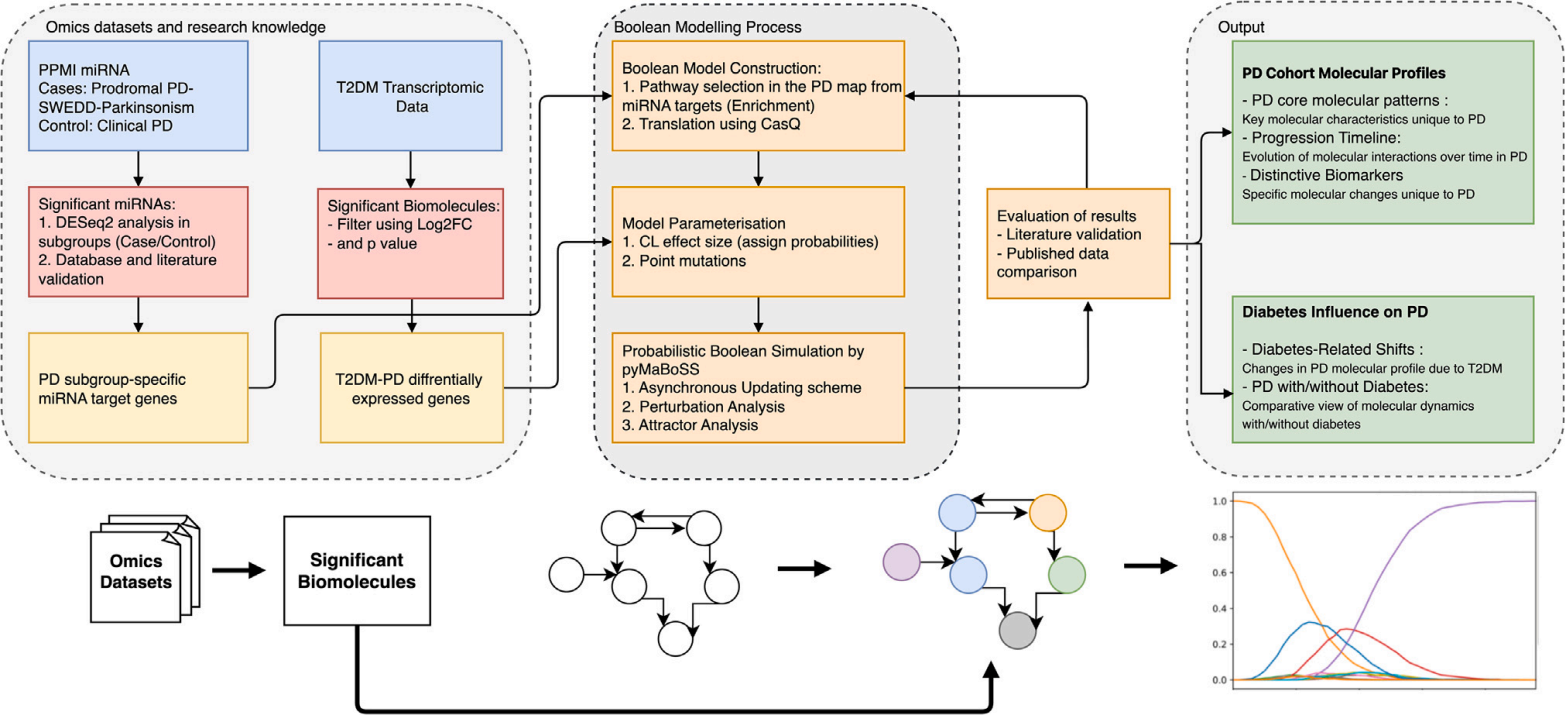
Research Workflow for Boolean modeling Parkinson’s Disease map and data integration This diagram outlines the research framework for understanding the molecular interactions in Parkinson’s disease groups. We integrate omics datasets and research knowledge to identify significant biomolecules, construct a Boolean model for simulation, and evaluate the results to determine PD cohort molecular profiles and the influence of diabetes on PD progression.

#### Parkinson’s Progression Markers Initiative-miRNAs dataset

The Parkinson’s Progression Markers Initiative (PPMI) dataset constitutes a longitudinal observational study of multiple disease cohorts. From the PPMI dataset we used microRNAs derived from blood samples of individuals from cohorts as listed below.

(1) **Clinical PD:** Describes a group of 1,430 individuals who exhibit clinical manifestations of PD and possess a positive dopamine transporter (DAT) SPECT scan.
(2) **Prodromal PD:** Denotes individuals whose symptoms have not yet severely manifested, yet their DAT SPECT results are significantly positive, a group that includes 223 individuals in this dataset.
(3) **SWEDD:** Describes individuals who, despite a clinical diagnosis of PD, do not exhibit a dopaminergic deficit on their DAT SPECT scan, encompassing 187 individuals in this dataset.
(4) **Atypical Parkinson’s Disease/Parkinsonism:** Characterizes a group of 81 individuals who exhibit idiopathic symptoms similar to those observed in standard PD.

This dataset is accessible through the Laboratory of Neuro Imaging (LONI) archive at www.ppmi-info.org/data.

#### Analysis of PPMI dataset

We analyzed miRNA expression data from the PPMI dataset, focusing on differentially expressed miRNAs between case and control cohorts. The analysis was conducted using R, a statistical programming language, with specific emphasis on the DESeq2 package (RRID_000154) for processing and analysing count-based NGS (next-generation sequencing) data. The methodology was structured as follows: The DESeq2 package in R was used to calculate the log2 fold change (log2FC) in miRNA expression between the case and control groups. DESeq2 employs a parametric approach, assuming that the count data follows a negative binomial distribution, which is appropriate for RNA-seq data characterised by overdispersion. We calculated normalised expression values and log2FC values to identify miRNAs with significant changes in expression levels between the cohorts.^141^ Further, *p*-values were calculated using the paired sample t-test, allowing us to account for paired observations between case and control cohorts.^142^ Given the multiple comparisons inherent in miRNA expression studies, we implemented the False Discovery Rate (FDR) correction, specifically the Benjamini-Hochberg procedure, to control for type I errors and reduce the likelihood of false positives. Therefore, we can identify truly significant miRNAs while controlling the proportion of false discoveries.

We then identified validated miRNA targets utilizing manually curated databases, with miRTarBase serving as a prominent resource.^143^ miRTarBase encompasses over three hundred and sixty thousand experimentally validated miRNA-target interactions. In addition to the miRTarBase, we considered other databases to identify the consensus of miRNA target interactions. These datasets included DIANA-TarBase, miRanda, PicTar (RRID:SCR_003343) and TargetScan (RRID:SCR_010845) which include experimentally validated data with high quality annotation. We filtered miRNA-target interactions by applying a threshold of experimental evidence level ‘A’ in miRTarBase and considered only interactions that were supported by at least two independent studies in the other databases. The overlapped miRNA target interactions were selected for our analysis.

Identified miRNA targets were filtered to match the expression profile of PD substantia nigra. To this end, we only retained miRNAs having at least one target among the SN-expression profile. This profile contains differentially expressed genes from a meta-analysis of eight transcriptomic profiles of postmortem substantia nigra of PD patients vs. controls,^34^ which appear at least once in the PD map.

To further characterize these miRNAs, we compared their expression patterns with curated miRNA expression databases: MiREDiBase,^144^ miRGate,^145^ the Human miRNA and Disease Database (HMDD),^146^ and through GEO data screening.^147^

#### Calculation of the transcriptomic profile from an organoid model of type two diabetes mellitus

To examine the effect of T2DM on the progression of PD subtypes identified in the PPMI study, we used two transcriptomic datasets that elaborate on PINK1 and GBA mutations in T2DM.^148^

In the first dataset, samples from three distinct PD patients carrying a homozygous Q456X mutation in the PINK1 gene were analyzed in comparison to their respective isogenic gene-corrected controls. The dataset comprises differentially expressed genes (DEGs) identified through RNA-sequencing from iPSC-derived neurons after 30 days of differentiation. PINK1 mutant neurons show reduction of IRS1 levels and impaired insulin signaling, indicated by decreased phosphorylation of AKT at S473 and T308. The second dataset elucidates DEGs of the GBA N307S mutation, derived from isogenic control midbrain organoids. The GBA N307S mutation in midbrain organoids is primarily associated with impaired neuron differentiation and cell cycle defects. This mutation shows a significant alteration in lipid metabolism and insulin signaling. We validated the significance of these datasets for the study of PD-T2DM comorbidity as follows.

#### Enrichment analysis

We performed Gene Set Enrichment Analysis (GSEA), utilizing the MINERVA GSEA Plugin,to identify significant pathways within the Parkinson’s Disease (PD) map. Our selection criteria was based on pathways showing signs of insulin resistance and metabolic dysregulations, key elements in PD pathology.^149^ Additionally, we used the MsigDB Hallmark 2020 database to identify the molecular signatures relevant to the datasets. These signatures were chosen based on their significant *p*-values and the relevance of their names known to be associated PD. Further, we performed pathway enrichment analysis using the EnrichNet tool. This tool highlights significant pathways based on significant *p*-values. Furthermore, we analyzed the brain tissue specificity using InnateDB (RRID:SCR_006714). Based on the complete set of DEGs, we calculated the XD score metric with a stringent threshold of 0.5, which represents the degree to which a DEG is expressed in a specific tissue type. XD (Cross-Domain) measures the similarity of gene sets by comparing them across different biological contexts or domains, such as tissue types, diseases, or functional categories, to identify commonalities and differences in their network-based relationships.

#### Literature review

A literature search was conducted to find validated miRNA targets commonly found in both PD and DM. Articles that reported such findings were reviewed and the relevant information was extracted. The results from this literature search were further filtered by the significant targets identified through the enrichment analysis.

#### Comparative analysis

The filtered common miRNA targets were compared with those identified in the PPMI dataset. The aim was to establish a connection between the two diseases prior to proceeding with simulation.

#### Constructing boolean models from systems biology diagrams

We constructed Boolean models based on system biology diagrams from the Parkinson’s disease map hosted on the MINERVA Platform.^7^ The MINERVA Platform provides the capacity to export selected segments of the map, and we refer to these parts as diagrams. We implemented a stratification process based on diagrams selected through a pathway enrichment analysis of the PPMI dataset using the Parkinson’s disease map. Once we identified significant pathways, we exported these as diagrams in CellDesigner (RRID:SCR_007263) SBML formats for subsequent modeling.

We then converted these models into SBML-qual, a designated module of the SBML standard specifically crafted to represent qualitative models of biological systems. To translate these diagrams into SBML-qual models, we used CaSQ (CellDesigner as SBML-qual).^150^ We transformed the diagrams into the Simple Interaction Format (SIF) with CaSQ, to create SBML-qual models that are compatible with tools such as BoolNet.^151^

#### Probabilistic boolean model simulation

The selected Boolean Models (BMs) were simulated using the pyMaBoSS framework, a Python (RRID:SCR_024202) API designed for the MaBoSS software^25^ for probabilistic Boolean modeling and simulation. pyMaBoSS leverages continuous time Markov processes. It utilizes a Monte Carlo algorithm for simulating the system’s evolution over time, based on the initial states of the biomolecules and the governing rules of their interactions. pyMaBoSS employs asynchronous updates in a random walk manner, updating a state of a single biomolecule at each step. Random asynchronous transitions are applied within pyMaBoSS to discover steady states and complex attractors from predefined initial states. In this simulation process, the probabilistic transitions were parameterized based on specific biological rules derived from experimental data. Each node (representing a biomolecule) was assigned an initial state determined by the input from the PPMI miRNA dataset, with probabilistic rules governing the state transitions reflecting the dynamics of molecular interactions within PD pathology. This allowed us to capture both deterministic and stochastic behaviors in disease progression In the probabilistic Boolean simulation, each condition was analyzed with 100 iterations and 1000 repetitions to ensure a robust representation of dynamic alterations. The high-performance computing (HPC) facility was used, specifically the Aion cluster with Intel Xeon Gold processors (20 cores, 192 GB RAM per node), was used for these simulations. This setup confirmed the consistency of our findings and provided a comprehensive view of the system’s dynamics.

#### Parametrization using PPMI miRNA data

To parameterise the constructed models, we estimated the size of effects for each microRNA using the Cohen distance.^152^ This measure provides a standardized mean difference between two distinct groups, taking into account the standard deviation. The calculated Cohen distances were used as a proxy for quantifying the biological impact of miRNA expression changes between case and control groups. The greater the distance, the more likely that miRNA had a substantial role in influencing disease progression dynamics. To make these findings more comprehensible, we converted the Cohen distance into probability values using the Common Language effect size (CL) method, as detailed in works by Ruscio^153^ and McGraw.^154^ The translated probabilities derived from the Cohen distance were assigned to the initial states of the corresponding miRNA targets within the SBML qual models. These computed probabilities reflect the conditions of various disease subtypes.

Simulations with random walks across probabilistic Boolean Models (BMs) were performed with pyMaBoSS to determine the likelihood of the outcomes (phenotypes) observed from these models. Each simulation run produced trajectories that allowed us to model disease pathways under different perturbations, giving us insights into the potential outcomes of various molecular interactions within the PD subtypes and in comorbidity scenarios.This allowed to examine how certain molecular alterations can affect the likelihood of different disease phenotypes. To identify significant shifts within these simulations, we used a regression technique for detecting multiple change points, as outlined by Lindelov.^155^

#### Parametrization using T2DM transcriptomic profile

The biomolecules of the model were parameterized based on T2DM transcriptomic expression profiles. The conversion of expression profiles into perturbations was achieved by categorizing changes in gene expression as either knockouts or overexpressions. A knockout perturbation involved setting the expression level of a biomolecule to “zero” indicating a complete loss of function. An overexpression perturbation was represented by setting the expression level to “one” indicating an increased activity beyond its normal physiological level. The application of knockouts and overexpressions was derived from the two datasets pertaining to mutations in the PINK1 and GBA1 genes. The knockouts and overexpressions were incorporated into the Boolean models as point mutations to simulate specific genetic alterations. The “point mutation” term refers to in silico targeted modifications in the biomolecules of the model. These targeted alterations are permanent changes in the state of the model biomolecules. The T2DM transcriptomic profile is independent from the PPMI subgroups. We aimed to understand the in silico impact of PINK1 on model behavior in comorbidity cases. Although patients with a PINK1 mutation in the T2DM model do not directly match specific clinical subtypes of PPMI cohorts, the underlying molecular mechanisms - such as mitochondrial dysfunction and insulin resistance - are pertinent to both conditions. This enables us to simulate and explore the potential effects of T2DM-related genetic mutations on PD progression.

#### Comparison of simulation trajectories

To quantify and compare two simulaton trajectories, we used Dynamic Time Warping (DTW).^156^ DTW operates by dividing the time series into points and measuring the distance between corresponding points in different series (Figure). In this context, DTW was used to quantify variability of simulation trajectories based on calculated change points. DTW calculates the distance between each point in one series and every point in the other series, identifying the optimal path that minimizes the total distance between the series. A lower DTW score indicates a higher similarity between the series(Limitations of the study). A lower DTW score suggests greater similarity between series. Thus, the DTW score can be used as a measure of the “activity” or dynamics of a particular process or trajectory over time. A lower DTW score could suggest a higher level of activity or more consistent condition progression, while a higher DTW score could indicate less activity or more variability in the process progression. However, interpreting the DTW score requires an understanding of the specific characteristics of the disease conditions. Pearson correlation was utilized to measure the correlation between DTW similarity values in pairs of subgroups (Table 12).

**Figure:**
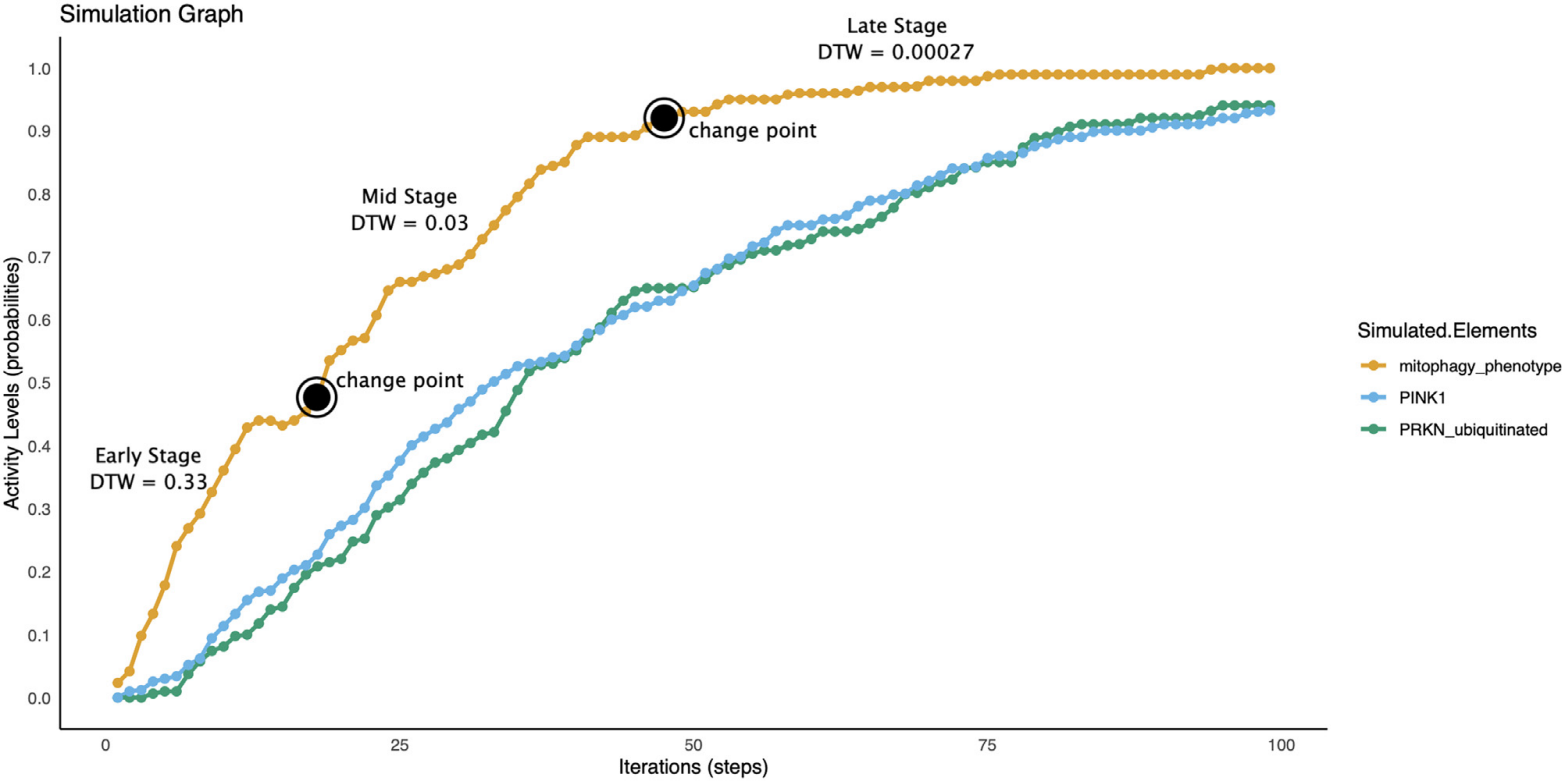
Dynamic Time Warping in PRKN Mitophagy Simulation The figure shows a representative run of the simulation in the PRKN mitophagy model, consisting of 100 iteration steps with 100 repetitions. Dynamic time warping measures the similarity between two sequences, which differs in speed based on different stages of the simulation (early, mid, and late).

### Quantification and statistical analysis

Various statistical analyses were performed to evaluate the significance of experimental results and ensure the robustness of the data. Below, we provide details on the statistical methods, software used, and where the corresponding statistical information can be found (e.g., figure legends, results sections).

#### Statistical software

(1) **R (RRID:****SCR_001905****)**: All statistical computing was performed using the R programming environment (v4.1.0). We employed packages such as *DESeq2* (RRID:SCR_000154) for RNA-seq data analysis and *ggplot2* (RRID:SCR_014601) for visualization.
(2) **Python (RRID:****SCR_008394****)**: Python libraries such as *NumPy* (RRID:SCR_008633) and *SciPy* (RRID:SCR_008058) were used for statistical tests and data manipulation. Boolean modeling simulations were conducted using *pyMaBoSS* (RRID:SCR_024202).
(3) **MaBoSS (v2.0)**: The MaBoSS software was used for probabilistic Boolean modeling simulations.
(4) **GSEA and EnrichNet**: Gene Set Enrichment Analysis (GSEA) and pathway enrichment analyses were performed using *EnrichNet* (RRID:SCR_005659) and *MSigDB* (RRID:SCR_016863).

#### Statistical tests and corrections

(1) **Differential expression analysis**: For miRNA expression data, the *DESeq2* package was employed to calculate $\log_{2}$ fold changes ( $\log_{2}$ FC) between case and control groups. Statistical significance was assessed using the Wald test, and the *Benjamini-Hochberg* False Discovery Rate (FDR) procedure was applied to correct for multiple testing, with an FDR threshold of 0.05 considered significant.
  – *Exact value of n*: The value of *n* represents the number of individuals per cohort as indicated in the figure legends (Clinical PD: n=1430, Prodromal PD: n=223, SWEDD: n=187, Atypical Parkinsonism: n=81).
(2) **Effect size calculations**: Effect sizes were computed using *Cohen’s d* to estimate the standardized mean difference between groups. The results were then translated into probability values using the *Common Language effect size* (CL) method.
(3) **Dynamic Time Warping (DTW) and regression**: DTW was employed to compare simulation trajectories between PD subtypes (Figure). The distance between each trajectory was computed using DTW, and *Pearson correlation* was used to assess the similarity between subgroups. Regression techniques were applied to detect multiple change points in the time series data, as per the method by Lindelov.

#### Dispersion and precision measures

(1) **Measures of central tendency and dispersion**: Data are reported as *mean* ± *standard deviation* (SD) or *median* with *interquartile ranges* (IQR), depending on data distribution. These are provided in the relevant figure legends and supplementary materials.
(2) **Confidence intervals**: 95% *confidence intervals* (CIs) were calculated for fold changes and effect sizes. These are provided alongside the relevant figures.

#### Sample sizes

The sample size (*n*) is explicitly noted in methods and results sections, where it represents the number of individuals per cohort, cells in culture, or biological replicates (e.g., Clinical PD: n=1430, Prodromal PD: n=223, etc.).

#### Statistical assumptions

(1) **Normality assumptions**: Assumptions of normality for parametric tests (e.g., t-tests) were verified using the *Shapiro-Wilk test* and visual inspection of Q-Q plots.
(2) **Overdispersion**: In RNA-seq analyses, the assumption of a negative binomial distribution was made for count-based data, with *DESeq2* accounting for overdispersion.

All additional statistical details, including the exact value of *n*, statistical tests used, and significance thresholds, are described in the figure legends and methods. Results are presented with appropriate measures of central tendency and dispersion, along with precise confidence intervals.
